# Molecular Targets of Breast Cancer: AKTing in Concert

**DOI:** 10.4137/bcbcr.s787

**Published:** 2008-05-23

**Authors:** Alakananda Basu

**Affiliations:** Department of Molecular Biology and Immunology, University of North Texas Health Science Center, Fort Worth, Texas, 76107

**Keywords:** Akt, PTEN, mTOR, breast cancer therapy

## Abstract

Despite significant advancement in the diagnosis and treatment of breast cancer, many patients succumb to this disease. The elucidation of aberrant signaling pathways that lead to breast cancer should help develop more effective therapeutic strategies. The Akt signaling pathway plays an important role in the development and progression of breast cancer. Overexpression/activation of Akt has been associated with poor prognosis and resistance to hormonal and chemotherapy. Although mutations in Akt are rare in breast cancer, the activity of Akt is regulated by hormones, growth factors, growth factor receptors, oncogenes and tumor suppressor genes that are often deregulated in breast cancer. The objective of this commentary is to discuss recent literature on how activation of Akt by various signaling pathways contributes to breast cancer and confers resistance to current therapy.

## Introduction

One in four women is diagnosed with breast cancer, which is the second leading cause of cancer-related death in women in the United States. Current treatment options include surgery, radiation and systemic therapy (e.g. hormonal therapy, chemotherapy and biological therapy). Major problems with chemotherapy are toxic side effects and emergence of drug resistance. The development of rational therapeutic approaches for breast cancer requires an understanding of the cellular signaling pathways leading to breast cancer. Since estrogen promotes growth of breast cancer and 70% of breast cancer patients are estrogen receptor (ER)-positive, antiestrogens (e.g. tamoxifen) are frequently used to treat patients with breast cancer. Fulvestrant, a novel ER antagonist that degrades ER, is more effective than tamoxifen (Raina, 2004). Aromatase inhibitors (e.g. letrozole, anastrozole and exemestane), which inhibit estrogen synthesis, are now in use to treat postmenopausal women with breast cancer. The use of antiestrogens and aromatase inhibitors is, however, recommended for hormone receptor-positive breast tumors. In addition, inherent and acquired resistance to endocrine therapy is a significant problem (Gururaj et al. 2006).

**Figure 1.**
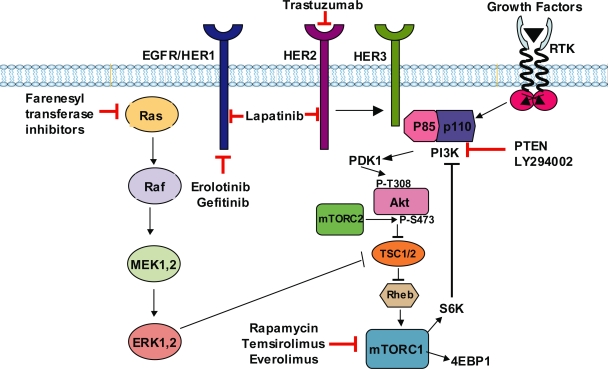
Akt signaling and targets for breast cancer therapy.

The human epidermal growth factor receptor (EGFR) or HER family of proteins comprised of EGFR (HER1), HER2, HER3 and HER4 are type 1 tyrosine kinase growth factor receptors (Arteaga, 2003; Gullick and Srinivasan, 1998). *HER2* gene is amplified or overexpressed in up to 25% of patients with breast cancer (Slamon et al. 1987). It can contribute to both development of breast cancer and its progression to aggressive forms. Herceptin (trastuzumab), a humanized monoclonal antibody against HER2/neu, was the first HER-2-targeted biologic agent approved for the therapy of breast cancer (Fig. 1). Although trastuzumab improved the survival of patients with HER2-positive metastatic breast cancers, especially when combined with chemotherapy, the development of resistance to trastuzumab and its high cost restrict its therapeutic use. Several small molecule inhibitors that target EGFR tyrosine kinase activity, (e.g. erlotinib, gefitinib, lapatinib and canertinib), farnesyl transferase inhibitors that target Ras, and rapamycin and its analogues that inhibit mammalian target of rapamycin (mTOR) have been developed (Fig. 1). These compounds show limited success as a single agent but they are more effective when combined with trastuzumab, antiestrogens or aromatase inhibitors (Gligorov et al. 2007; Goss and Wu, 2007).

The development of successful therapeutic agents requires not only identification of appropriate molecular targets but also elucidation of the signaling pathways that may contribute to resistance to these agents. The Akt/protein kinase B (PKB) serine/threonine kinase regulates a myriad of cellular processes, including metabolism, cell proliferation, cell survival and cell growth (Vivanco and Sawyers, 2002). It mediates its multiple functions by phosphorylating a large number of substrates, including glycogen synthase kinase-3, cyclin D1, cyclin-dependent kinase inhibitors (p27 and p21), caspase-9, Bad, MDM2 and forkhead transcription factors (Woodgett, 2005; Liu et al. 2007; Vivanco and Sawyers, 2002). Activation of Akt has been associated with up to 40% of breast cancers (Liu et al. 2007). The increase in phospho-Akt level in breast tumor samples correlates with poor prognosis (Stal et al. 2003; Tokunaga et al. 2006; Zhou et al. 2004) and predicts a worse outcome among endocrine-treated patients (Perez-Tenorio, 2002). Akt has been associated with tumor progression, invasion, metastasis, and resistance to hormonal and chemotherapy (DeGraffenried et al. 2003; Faridi et al. 2003; Liu et al. 2007; Qiao et al. 2007; Sale and Sale, 2007; Zhao et al. 2007). Thus, Akt is an important therapeutic target for the treatment of breast cancer. This commentary discusses how deregulation in various signaling pathways contributes to breast cancer via activation of the Akt signaling pathway.

## Activation of Akt

Although many breast tumors exhibit an increase in constitutively-active Akt, mutations in Akt are rare, suggesting that the elevated Akt activity results from an alteration of regulators of Akt rather than any mutation (Sun, 2001b). Akt is activated by a variety of growth factors, including insulin, insulin-like growth factor-1 and epidermal growth factor (Sale and Sale, 2007). It acts downstream of phosphoinositide-3 kinase (PI3K) and mediates the survival effects of different growth factors, cytokines and oncogenes (Hemmings, 1997). Activation of PI3K generates 3-phosphoinositides that bind to the pleckstrin homology (PH) domain of Akt inducing its membrane translocation and conformational change required for its phosphorylation and activation (Galetic, 1999). Phosphorylation appears to be critical for its kinase activity (Chan, 1999; Galetic, 1999). Akt is phosphorylated at Thr308 in the activation loop by phosphoinositide-dependent protein kinase 1 (PDK1) (Alessi et al. 1997, Williams et al. 2000). Phosphorylation of Akt at the Ser473 site in the carboxy terminal domain is necessary for its full activation. Several kinases, including PDK1, integrin-linked kinase (ILK) (Delcommenne et al. 1998; Troussard et al. 2003), rictor-mTOR complex (Sarbassov et al. 2005), mitogen-activated protein kinase-activated protein kinase 2 (MAPKAP kinase-2) (Alessi et al. 1996; Rane, 2001) and DNA-dependent protein kinase (DNA-PK) (Feng et al. 2004) have been implicated in phosphorylating the Ser473 site in Akt.

## The Involvement of Akt Isoforms in Breast Cancer

Three isoforms of Akt have been identified: Akt1/PKBα, Akt2/PKBβ and Akt3/PKBγ (Manning and Cantley, 2007). Akt1 is the most abundant isoform. All three Akt isoforms have been associated with breast cancer (Bacus et al. 2002; Bellacosa et al. 1995; Nakatani et al. 1999; Perez-Tenorio and Stal, 2002; Stal et al. 2003; Sun et al. 2001a; Sun et al. 2001b; Faridi et al. 2003; Ju et al. 2007). Akt1 is overexpressed in breast cancer cells and has been shown to be important in estrogen-stimulated growth (Ahmad et al. 1999; Stal et al. 2003). It has been reported that Akt2 is amplified in 3% of breast cancers (Bellacosa, 1995) and is frequently activated in primary human breast carcinoma (Sun, 2001a). Akt1 and Akt2 have opposite role in invasion and metastasis. While Akt1 inhibits invasion, metastasis and epithelial to mesenchy-mal transition (EMT), Akt2 facilitates cell migration and EMT (Irie et al. 2005; Toker and Yoeli-Lerner, 2006). A recent report suggests that Akt2 contributes to breast cancer metastasis via protein kinase C (PKC)-ζ (Wang et al. 2008). Upregulation of Akt3 may contribute to the more aggressive clinical phenotype of the ER-negative breast cancers (Nakatani K, 1999) although one report showed Akt3 mRNA expression is not restricted to tumorigenic cell lines or to ER-negative breast cancer cells (Zinda, 2001). However, transfection of constitutively active Akt-3 in MCF-7 cells reversed estrogen and tamoxifen response of MCF-7 cells (Faridi et al. 2003). Thus, an understanding of the distinct role of Akt isoforms is essential to exploit the Akt signaling pathway for breast cancer therapy.

## Akt Takes Center Stage

Several signaling pathways that are deregulated in breast cancer act via the Akt signaling pathway. Akt is often activated in breast tumors overexpressing HER2 (Tokunaga et al. 2006). It has been reported that HER2 mediates its oncogenic signaling via the PI3K/Akt signaling pathway (Hsieh and Moasser, 2007). Although HER2 does not directly bind to and activate PI3K/Akt, it activates Akt through tyrosine phosphorylation of HER3 (Soltoff et al. 1994). HER2 forms heterodimers with HER3 and transphosphorylation of HER3 by HER2 allows HER3 to interact with the p85 regulatory subunit of PI3K (Fig. 1), thereby causing activation of PI3K/Akt (Soltoff et al. 1994). It has been shown that the p85 regulatory subunit of PI3K can also interact with ERα causing activation of Akt (Simoncini et al. 2000). Furthermore, PI3K can cause phosphorylation and activation of ER. Thus, a combination of PI3K/Akt inhibitors with trastuzumab, tyrosine kinase inhibitors and antiestrogens may be more effective in treating breast cancer compared to a single agent.

Since Akt activity is regulated by phosphorylation at the Ser473 site, a deregulation in kinases and phosphatases that regulate phosphorylation at this site can result in activation of Akt. Integrin-linked kinase (ILK) has been shown to phosphorylate Akt at the S473 site although it is not clear if ILK directly phosphorylates Akt or if it acts as a scaffolding protein allowing another kinase to phosphorylate Akt (Hinton et al. 2008). Recently, it has been reported that a rictor/ILK complex regulates phosphorylation of Akt at Ser473 (McDonald et al. 2008). ILK has been associated with breast cancer progression and metastasis through Akt activation (Hinton et al. 2008).

The tumor suppressor protein phosphatase and tensin homolog deleted on chromosome 10 (PTEN) that dephosphorylates the 3 position of phosphoinositides and thereby inactivates Akt is also deregulated in breast carcinoma (Cantley, 1999). Although mutations in PTEN have been detected in a small percentage of breast cancers, loss of heterozygosity (LOH) at the PTEN locus is a more common occurrence in breast cancer (Haiman et al. 2006). The reduced expression of PTEN protein was found in 30% of breast cancers and correlated with lymph node metastases and a worse prognosis (Tsutsui et al. 2005). The PH domain/leucin-rich repeat protein phosphatase (PHLPP) that directly dephosphorylates Ser473 in Akt and thereby inactivates it has recently been identified (Gao et al. 2005). A decrease in the expression of PHLPP was associated with progression to metastasis in a series of human epithelial cells obtained by successive biopsies from one breast cancer patient (Qiao et al. 2007).

Akt can act both upstream and downstream of mammalian target of rapamycin (mTOR) which regulates protein translation and cell cycle progression via its downstream effectors p70 S6 kinase (p70S6K) and 4E-binding protein-1 (Fig. 1). mTOR can form a complex with either raptor or rictor to form mTORC1 or mTORC2, respectively (Sarbassov et al. 2005). mTORC1, but not mTORC2, is rapamycin sensitive. Akt can activate mTOR by phosphorylating and inactivating the tumor suppressor protein tubersclerosis complex 2 (TSC2), which negatively regulates mTOR activity. Furthermore, persistent activation of p70S6K can lead to feedback inhibition of the PI3K/Akt pathway (O’Reilly et al. 2006). mTOR is critical for the proliferative responses mediated by growth factor receptors (Mita et al. 2003) and is required for estrogen-induced breast tumor cell proliferation (Boulay et al. 2005). Constitutive signaling through this pathway is a cause of treatment failure in breast cancer patients (Crowder and Ellis, 2005). Rapamycin and its analogues, such as temsirolimus (CCI-779) and everolimus (RAD-001) are in clinical trials (Fig. 1). Breast cancer cells with activated Akt are exquisitely sensitive to mTOR antagonists (Yu et al. 2001). Activation of Akt/mTOR pathway can cause resistance to tamoxifen (Clark et al. 2002). Combination of mTOR antagonists with antiestrogens and aromatase inhibitors has been effective in treating hormone-sensitive breast cancers (deGraffenried et al. 2004). A recent study showed that mutation perturbing the lipid and protein phosphatase activities of PTEN caused activation of Akt/mTOR/p70S6K signaling and chemoresistance, whereas rapamycin synergized with doxorubicin to inhibit growth of breast cancer cells (Steelman et al. 2008).

## Conclusion

The signaling pathways that are altered in breast cancer or in response to therapy can greatly influence clinical outcome. The development of molecular-targeted therapies presents promises as well as challenges. The PI3K/Akt signaling pathway has received considerable attention because of its critical role in the development and progression of breast cancer. In addition, the status of Akt can influence both inherent and acquired resistance to systemic therapy. In targeting the Akt pathway, one must consider that different Akt isoforms may have redundant, distinct or opposite functions. In addition, the status of different signaling pathways that feed into the Akt signaling pathway may have a significant impact on patient response to therapy. Thus, while combining inhibitors of the PI3K/Akt pathway in conjunction with currently available therapeutic agents may greatly improve their efficacy, finding the right combination is the challenge.

## References

[b1-bcbcr-2008-011] Ahmad S, Singh N, Glazer RI (1999). Role of AKT1 in 17beta-estradiol- and insulin-like growth factor I (IGF-I)-dependent proliferation and prevention of apoptosis in MCF-7 breast carcinoma cells. Biochem. Pharmacol.

[b2-bcbcr-2008-011] Alessi DR, Andjelkovic M, Caudwell B, Cron P, Morrice N, Cohen P, Hemmings BA (1996). Mechanism of activation of protein kinase B. by insulin and IGF-1. Embo. J.

[b3-bcbcr-2008-011] Alessi DR, James SR, Downes CP, Holmes AB, Gaffney PR, Reese CB, Cohen P (1997). Characterization of a 3-phosphoinositide-dependent protein kinase which phosphorylates and activates protein kinase Balpha. Curr. Biol.

[b4-bcbcr-2008-011] Arteaga C (2003). Targeting HER1/EGFR: a molecular approach to cancer therapy. Semin. Oncol.

[b5-bcbcr-2008-011] Bacus SS, Altomare DA, Lyass L, Chin DM, Farrell MP, Gurova K, Gudkov A, Testa JR (2002). AKT2 is frequently upregulated in HER-2/neu-positive breast cancers and may contribute to tumor aggressiveness by enhancing cell survival. Oncogene.

[b6-bcbcr-2008-011] Bellacosa A, De Feo D, Godwin AK, Bell DW, Cheng JQ, Altomare DA, Wan M, Dubeau L, Scambia G, Masciullo V, Ferrandina G, Benedetti Panici P, Mancuso S, Neri G, Testa JR (1995). Molecular alterations of the AKT2 oncogene in ovarian and breast carcinomas. Int. J. Cancer.

[b7-bcbcr-2008-011] Boulay A, Rudloff J, Ye J, Zumstein-Mecker S, O’reilly T, Evans DB, Chen S, Lane HA (2005). Dual inhibition of mTOR and estrogen receptor signaling in vitro induces cell death in models of breast cancer. Clin. Cancer Res.

[b8-bcbcr-2008-011] Cantley LC, Neel BG (1999). New insights into tumor suppression: PTEN suppresses tumor formation by restraining the phosphoinositide 3-kinase/Akt pathway. Proc. Natl. Acad. Sci. USA.

[b9-bcbcr-2008-011] Chan TO, Rittenhouse SE, Tsichlis PN (1999). Akt/PKB. and other D3 phosphoinositide-regulated kinases: kinase activation by phosphoinositide-dependent phosphorylation. Annu. Rev. Biochem.

[b10-bcbcr-2008-011] Clark AS, West K, Streicher S, Dennis PA (2002). Constitutive and inducible Akt activity promotes resistance to chemotherapy, trastuzumab, or tamoxifen in breast cancer cells. Mol. Cancer Ther.

[b11-bcbcr-2008-011] Crowder RJ, Ellis MJ (2005). Treating breast cancer through novel inhibitors of the phosphatidylinositol 3′-kinase pathway. Breast Cancer Res.

[b12-bcbcr-2008-011] Degraffenried LA, Friedrichs WE, Fulcher L, Fernandes G, Silva JM, Peralba JM, Hidalgo M (2003). Eicosapentaenoic acid restores tamoxifen sensitivity in breast cancer cells with high Akt activity. Ann. Oncol.

[b13-bcbcr-2008-011] Degraffenried LA, Friedrichs WE, Russell DH, Donzis EJ, Middleton AK, Silva JM, Roth RA, Hidalgo M (2004). Inhibition of mTOR activity restores tamoxifen response in breast cancer cells with aberrant Akt Activity. Clin. Cancer Res.

[b14-bcbcr-2008-011] Delcommenne M, Tan C, Gray V, Rue L, Woodgett J, Dedhar S (1998). Phosphoinositide-3-OH kinase-dependent regulation of glycogen synthase kinase 3 and protein kinase B/AKT by the integrin-linked kinase. Proc. Natl. Acad. Sci. USA.

[b15-bcbcr-2008-011] Faridi J, Wang L, Endemann G, Roth RA (2003). Expression of constitutively active Akt-3 in MCF-7 breast cancer cells reverses the estrogen and tamoxifen responsivity of these cells in vivo. Clin. Cancer Res.

[b16-bcbcr-2008-011] Feng J, Park J, Cron P, Hess D, Hemmings BA (2004). Identification of a PKB/Akt Hydrophobic Motif Ser-473 Kinase as DNA-dependent Protein Kinase. J. Biol. Chem.

[b17-bcbcr-2008-011] Galetic I, Andjelkovic M, Meier R, Brodbeck D, Park J, Hemmings BA (1999). Mechanism of protein kinase B activation by insulin/insulin-like growth factor-1 revealed by specific inhibitors of phosphoinositide 3-kinase-significance for diabetes and cancer. Pharmacol. Ther.

[b18-bcbcr-2008-011] Gao T, Furnari F, Newton AC (2005). PHLPP: a phosphatase that directly dephosphorylates Akt, promotes apoptosis, and suppresses tumor growth. Mol. Cell.

[b19-bcbcr-2008-011] Gligorov J, Azria D, Namer M, Khayat D, Spano JP (2007). Novel therapeutic strategies combining antihormonal and biological targeted therapies in breast cancer: focus on clinical trials and perspectives. Crit. Rev. Oncol. Hematol.

[b20-bcbcr-2008-011] Goss P, Wu M (2007). Application of aromatase inhibitors in endocrine responsive breast cancers. Breast.

[b21-bcbcr-2008-011] Gullick WJ, Srinivasan R (1998). The type 1 growth factor receptor family: new ligands and receptors and their role in breast cancer. Breast Cancer Res. Treat.

[b22-bcbcr-2008-011] Gururaj AE, Rayala SK, Vadlamudi RK, Kumar R (2006). Novel mechanisms of resistance to endocrine therapy: genomic and nongenomic considerations. Clin. Cancer Res.

[b23-bcbcr-2008-011] Haiman CA, Stram DO, Cheng I, Giorgi EE, Pooler L, Penney K, Le Marchand L, Henderson BE, Freedman ML (2006). Common genetic variation at PTEN and risk of sporadic breast and prostate cancer. Cancer Epidemiol Biomarkers Prev.

[b24-bcbcr-2008-011] Hemmings BA (1997). Akt signaling: Linking membrane events to life and death decisions. Science.

[b25-bcbcr-2008-011] Hinton CV, Avraham S, Avraham HK (2008). Contributions of Integrin-Linked Kinase to Breast Cancer Metastasis and Tumorigenesis. J. Cell. Mol. Med.

[b26-bcbcr-2008-011] Hsieh AC, Moasser MM (2007). Targeting HER. proteins in cancer therapy and the role of the non-target HER3. Br. J. Cancer.

[b27-bcbcr-2008-011] Irie HY, Pearline RV, Grueneberg D, Hsia M, Ravichandran P, Kothari N, Natesan S, Brugge JS (2005). Distinct roles of Akt1 and Akt2 in regulating cell migration and epithelial-mesenchymal transition. J. Cell Biol.

[b28-bcbcr-2008-011] Ju X, Katiyar S, Wang C, Liu M, Jiao X, Li S, Zhou J, Turner J, Lisanti MP, Russell RG, Mueller SC, Ojeifo J, Chen WS, Hay N, Pestell RG (2007). Akt1 governs breast cancer progression in vivo. Proc. Natl. Acad. Sci. U.S.A.

[b29-bcbcr-2008-011] Liu W, Bagaitkar J, Watabe K (2007). Roles of AKT signal in breast cancer. Front Biosci.

[b30-bcbcr-2008-011] Manning BD, Cantley LC (2007). AKT/PKB. signaling: navigating downstream. Cell.

[b31-bcbcr-2008-011] Mcdonald PC, Oloumi A, Mills J, Dobreva I, Maidan M, Gray V, Wederell ED, Bally MB, Foster LJ, Dedhar S (2008). Rictor and integrin-linked kinase interact and regulate Akt phosphorylation and cancer cell survival. Cancer Res.

[b32-bcbcr-2008-011] Mita MM, Mita A, Rowinsky EK (2003). Mammalian target of rapamycin: a new molecular target for breast cancer. Clin. Breast Cancer.

[b33-bcbcr-2008-011] Nakatani K, Thompson DA, Barthel A, Sakaue H, Liu W, Weigel RJ, Roth RA (1999). Up-regulation of Akt3 in estrogen receptor-deficient breast cancers and androgen-independent prostate cancer lines. J. Biol. Chem.

[b34-bcbcr-2008-011] O’reilly KE, Rojo F, She QB, Solit D, Mills GB, Smith D, Lane H, Hofmann F, Hicklin DJ, Ludwig DL, Baselga J, Rosen N (2006). mTOR inhibition induces upstream receptor tyrosine kinase signaling and activates Akt. Cancer Res.

[b35-bcbcr-2008-011] Perez-Tenorio G, Stal O (2002). Activation of AKT/PKB in breast cancer predicts a worse outcome among endocrine treated patients. Brit. J. Cancer.

[b36-bcbcr-2008-011] Qiao M, Iglehart JD, Pardee AB (2007). Metastatic potential of 21T human breast cancer cells depends on Akt/protein kinase B activation. Cancer Res.

[b37-bcbcr-2008-011] Raina V (2004). Is fulvestrant more effective than tamoxifen for treating ER-positive breast cancer in postmenopausal women. Nat. Clin. Pract. Oncol.

[b38-bcbcr-2008-011] Rane MJ, Coxon PY, Powell DW, Webster R, Klein JB, Pierce W, Ping P, Mcleish KR (2001). p38 kinase-dependent MAPKAPK-2 activation functions as 3-phosphoinositide-dependent kinase-2 for Akt in human neutrophils. J. Bio. Chem.

[b39-bcbcr-2008-011] Sale EM, Sale GJ (2007). Protein kinase B: signalling roles and therapeutic targeting. Cell Mol. Life Sci.

[b40-bcbcr-2008-011] Sarbassov DD, Guertin DA, Ali SM, Sabatini DM (2005). Phosphorylation and regulation of Akt/PKB by the rictor-mTOR complex. Science.

[b41-bcbcr-2008-011] Simoncini T, Hafezi-Moghadam A, Brazil DP, Ley K, Chin WW, Liao JK (2000). Interaction of oestrogen receptor with the regulatory subunit of phosphatidylinositol-3-OH kinase. Nature.

[b42-bcbcr-2008-011] Slamon DJ, Clark GM, Wong SG, Levin WJ, Ullrich A, Mcguire WL (1987). Human breast cancer: correlation of relapse and survival with amplification of the HER-2/neu oncogene. Science.

[b43-bcbcr-2008-011] Soltoff SP, Carraway KL, Prigent SA, Gullick WG, Cantley LC (1994). ErbB3 is involved in activation of phosphatidylinositol 3-kinase by epidermal growth factor. Mol. Cell. Biol.

[b44-bcbcr-2008-011] Stal O, Perez-Tenorio G, Akerberg L, Olsson B, Nordenskjold B, Skoog L, Rutqvist LE (2003). Akt kinases in breast cancer and the results of adjuvant therapy. Breast Cancer Res.

[b45-bcbcr-2008-011] Steelman LS, Navolanic PM, Sokolosky ML, Taylor JR, Lehmann BD, Chappell WH, Abrams SL, Wong EW, Stadelman KM, Terrian DM, Leslie NR, Martelli AM, Stivala F, Libra M, Franklin RA, Mccubrey JA (2008). Suppression of PTEN function increases breast cancer chemotherapeutic drug resistance while conferring sensitivity to mTOR inhibitors. Oncogene.

[b46-bcbcr-2008-011] Sun M, Paciga JE, Feldman RI, Yuan Z, Coppola D, Lu YY, Shel-ley SA, Nicosia SV, Cheng JQ (2001a). Phosphatidylinositol-3-OH kinase (PI3K)/Akt2, activated in breast cancer, regulates and is induced by estrogen receptor α (ERα) via interaction between ERα and PI3K. Cancer Res.

[b47-bcbcr-2008-011] Sun M, Wang G, Paciga JE, Feldman RI, Yuan ZQ, Ma XL, Shelley SA, Jove R, Tsichlis PN, Nicosia SV, Cheng JQ (2001b). AKT1/PKBalpha kinase is frequently elevated in human cancers and its constitutive activation is required for oncogenic transformation in NIH3T3 cells. Am. J. Pathol.

[b48-bcbcr-2008-011] Toker A, Yoeli-Lerner M (2006). Akt signaling and cancer: surviving but not moving on. Cancer Res.

[b49-bcbcr-2008-011] Tokunaga E, Kimura Y, Oki E, Ueda N, Futatsugi M, Mashino K, Yamamoto M, Ikebe M, Kakeji Y, Baba H, Maehara Y (2006). Akt is frequently activated in HER2/neu-positive breast cancers and associated with poor prognosis among hormone-treated patients. Int. J. Cancer.

[b50-bcbcr-2008-011] Troussard AA, Mawji NM, Ong C, Mui A, St -Arnaud R, Dedhar S (2003). Conditional knock-out of integrin-linked kinase demonstrates an essential role in protein kinase B/Akt activation. J. Biol. Chem.

[b51-bcbcr-2008-011] Tsutsui S, Inoue H, Yasuda K, Suzuki K, Higashi H, Era S, Mori M (2005). Reduced expression of PTEN protein and its prognostic implications in invasive ductal carcinoma of the breast. Oncology.

[b52-bcbcr-2008-011] Vivanco I, Sawyers CL (2002). The phosphatidylinositol 3-Kinase AKT pathway in human cancer. Nat. Rev. Cancer.

[b53-bcbcr-2008-011] Wang J, Wan W, Sun R, Liu Y, Sun X, Ma D, Zhang N (2008). Reduction of Akt2 expression inhibits chemotaxis signal transduction in human breast cancer cells. Cell. Signal.

[b54-bcbcr-2008-011] Williams MR, Arthur JS, Balendran A, Van Der Kaay J, Poli V, Cohen P, Alessi DR (2000). The role of 3-phosphoinositide-dependent protein kinase 1 in activating AGC kinases defined in embryonic stem cells. Curr. Biol.

[b55-bcbcr-2008-011] Woodgett JR (2005). Recent advances in the protein kinase B signaling pathway. Curr. Opin. Cell. Biol.

[b56-bcbcr-2008-011] Yu K, Toral-Barza L, Discafani C, Zhang WG, Skotnicki J, Frost P, Gibbons JJ (2001). mTOR, a novel target in breast cancer: the effect of CCI-779, an mTOR inhibitor, in preclinical models of breast cancer. Endocr Relat Cancer.

[b57-bcbcr-2008-011] Zhao M, Mueller BM, Discipio RG, Schraufstatter IU (2007). Akt plays an important role in breast cancer cell chemotaxis to CXCL12. Breast Cancer Res. Treat.

[b58-bcbcr-2008-011] Zhou X, Tan M, Stone Hawthorne V, Klos KS, Lan KH, Yang Y, Yang W, Smith TL, Shi D, Yu D (2004). Activation of the Akt/mammalian target of rapamycin/4E-BP1 pathway by ErbB2 overexpression predicts tumor progression in breast cancers. Clin. Cancer Res.

[b59-bcbcr-2008-011] Zinda M, Johnson MA, Paul JD, Horn C, Konicek BW, Lu ZH, Sandusky G, Thomas JE, Neubauer BL, Lai MT, Graff JR (2001). AKT-1, -2, and -3 are Expressed in Both Normal and Tumor Tisues of the Lung, Breast, Prostate, and Colon. Clin. Cancer. Res.

